# Diversity of *Eimeria* Species in Wild Chamois *Rupicapra* spp.: A Statistical Approach in Morphological Taxonomy

**DOI:** 10.3389/fvets.2020.577196

**Published:** 2020-10-14

**Authors:** Federica Berrilli, Margherita Montalbano Di Filippo, Claudio De Liberato, Ilaria Marani, Paolo Lanfranchi, Nicola Ferrari, Tiziana Trogu, Nicoletta Formenti, Francesco Ferretti, Luca Rossi, Stefano D'Amelio, Annunziata Giangaspero

**Affiliations:** ^1^Department of Clinical Sciences and Translational Medicine, Tor Vergata University, Rome, Italy; ^2^Istituto Zooprofilattico Sperimentale del Lazio e della Toscana “M. Aleandri”, Rome, Italy; ^3^Department of Veterinary Medicine, Università degli Studi di Milano, Milan, Italy; ^4^Istituto Zooprofilattico Sperimentale della Lombardia e dell'Emilia Romagna “Bruno Ubertini”, Brescia, Italy; ^5^Department of Life Sciences, University of Siena, Siena, Italy; ^6^Department of Veterinary Sciences, University of Turin, Grugliasco, Italy; ^7^Department of Public Health and Infectious Diseases, Sapienza University of Rome, Rome, Italy; ^8^Department of Science of Agriculture, Food and Environment, University of Foggia, Foggia, Italy

**Keywords:** *Eimeria* spp., *Rupicapra* spp., morphology, hierarchical clustering, Italy

## Abstract

Wildlife is frequently infected by intestinal protozoa, which may threaten their fitness and health. A diverse community of *Eimeria* species is known to occur in the digestive tract of mountain-dwelling ungulates, including chamois (genus *Rupicapra*). However, available data on *Eimeria* diversity in these taxa is at times inconsistent and mostly dated. In the present study, we aimed to revisit the occurrence of *Eimeria* spp. in the Alpine subspecies of the Northern chamois (*Rupicapra rupicapra rupicapra*) and the Apennine subspecies of the Southern chamois (*Rupicapra pyrenaica ornata*) in Italy, using an integrated approach based on a hierarchical cluster analysis (HCPC) applied to oocyst morphology and morphometry. A total of 352 fecal samples were collected from *R. r. rupicapra* (*n* = 262) and *R. p. ornata* (*n* = 90). Overall, 85.3% (300/352) of the animals tested microscopically positive to *Eimeria* spp. Based on morphological analysis, we identified all the eimerian species described in chamois. Through the HCPC method, five clusters were generated, corresponding to *E. suppereri, E. yakimoffmatschoulskyi, E. riedmuelleri* (two different clusters), and *E. rupicaprae* morphotypes. The well-defined clusters within *E. riedmuelleri* support the existence of two distinct morphological groups, possibly referable to different taxonomic units. This study suggests that combining a morphometrical approach with a powerful statistical method may be helpful to disentangle uncertainties in the morphology of *Eimeria* oocysts and to address taxonomic studies of eimeriid protozoa at a specific host taxon level.

## Introduction

Exploring the parasite communities in wild animals represents a main challenge for wildlife management, as several parasites may have an impact on their fitness and health, even more so in the frequent event of co-infections (1). Moreover, wildlife can play an important role as reservoirs of pathogens of medical and/or veterinary importance (2).

Wild caprines (Bovidae, Caprinae) are reportedly known to harbor rich parasite communities including representatives of the genus *Eimeria* Schneider, 1875 (Coccidia, Apicomplexa) (3). In particular, five *Eimeria* species have been described to infect the iconic members of the genus *Rupicapra*: *Eimeria alpina* Supperer and Kutzer, 1961; *Eimeria riedmuelleri* Yakimoff and Matschoulsky, 1940; *Eimeria rupicaprae* Galli-Valerio, 1924; *Eimeria suppereri* Kutzer, 1964 and *Eimeria yakimoffmatschoulskyi* Supperer and Kutzer, 1961 (4). These species have never been reported in other ruminants, thus advocating for their strict host specificity.

Traditionally, *Eimeria* species identification is obtained using a set of biological traits and morphological features such as the identity of the host species as well as oocyst size, shape and structure (curvature, presence/absence of oocyst residuum, conspicuous/inconspicuous micropyle), shape and structure of the sporocysts (5, 6). However, morphological methods are often challenging and several other *Eimeria* species from *Rupicapra* spp. have been inadequately or erroneously described. As stated in Levine and Ivens (4), the name *Eimeria longispora* Rudovsky 1922, identified in chamois from Austria, should be considered as *nomen nudum*, due to its incomplete description; moreover, the report of *Eimeria arloingi, Eimeria crandallis, Eimeria ninakohlyakimovae*, and *Eimeria parva* in *R. rupicapra* in the present-day Slovakia and of *E. arloingi* and *E. ninakohlyakimovae* in the present-day Slovenia (7, 8) should be considered uncertain; finally the occurrence of *Eimeria faurei* in hosts other than *Ovis* and *Capra* is doubtful.

In Italy, two taxa of chamois are present (9): the Alpine subspecies of the Northern chamois (*Rupicapra rupicapra rupicapra*), widely spread along the Alps, and the Apennine subspecies of the Southern chamois (*Rupicapra pyrenaica ornata*), which occurs in five protected areas of central Apennines. Due to its limited and fragmented distribution range as well as the small population size, *R. p. ornata* is currently included in the International Union for Conservation of Nature (IUCN) red list in the category of “vulnerable” taxa (VU D1+2) (www.iucn.it). Despite the relevance of *R. r. rupicapra* and *R. p. ornata*, which cover a large geographical area, data on *Eimeria* spp. in wild chamois from Italy are not only limited but also still leave open questions on their identity and prevalence. Indeed, in the Alpine chamois *R. r. rupicapra, E. rupicaprae* identification dates back to 1950's through a parasitological survey on the fauna of the Gran Paradiso National Park (Western Alps) (10) and about 20 years later, *E. rupicaprae, E. riedmuelleri*, and *E. yakimoffmatschoulskyi* were described in Eastern Alps (11). More recently, Stancampiano et al. (12) confirmed the presence of *E. riedmuelleri* and *E. yakimoffmatschoulskyi* in *R. r. rupicapra*, and recorded *E. suppereri* for the first time in Italy; more intriguingly, a coccidian species resembling *E. faurei*, a species related to domestic sheep was also described (12). As regards the Apennine chamois *R. p. ornata*, only one survey has been carried out in Italy, reporting *E. rupicaprae* and *E. riedmuelleri* (13). Furthermore, *E. alpina* and *E. yakimoffmatschoulskyi* have been recorded by Rossi et al. (14).

Despite the recent advances in morphological, morphometrical, statistical, and molecular biology-based approaches, which may be utilized to investigate the identity of coccidian oocysts, the use of a single methodology is unable to fully characterize these structures and different tools should be applied for taxonomic purposes (6). Therefore, in order to overcome the issues related to the traditional approach to taxonomy of *Eimeria*, the aim of this work was to combine morphological characterization of the oocysts with a statistical method to refine knowledge of *Eimeria* species in Alpine and Apennine chamois.

## Materials and Methods

**Figure 1:**
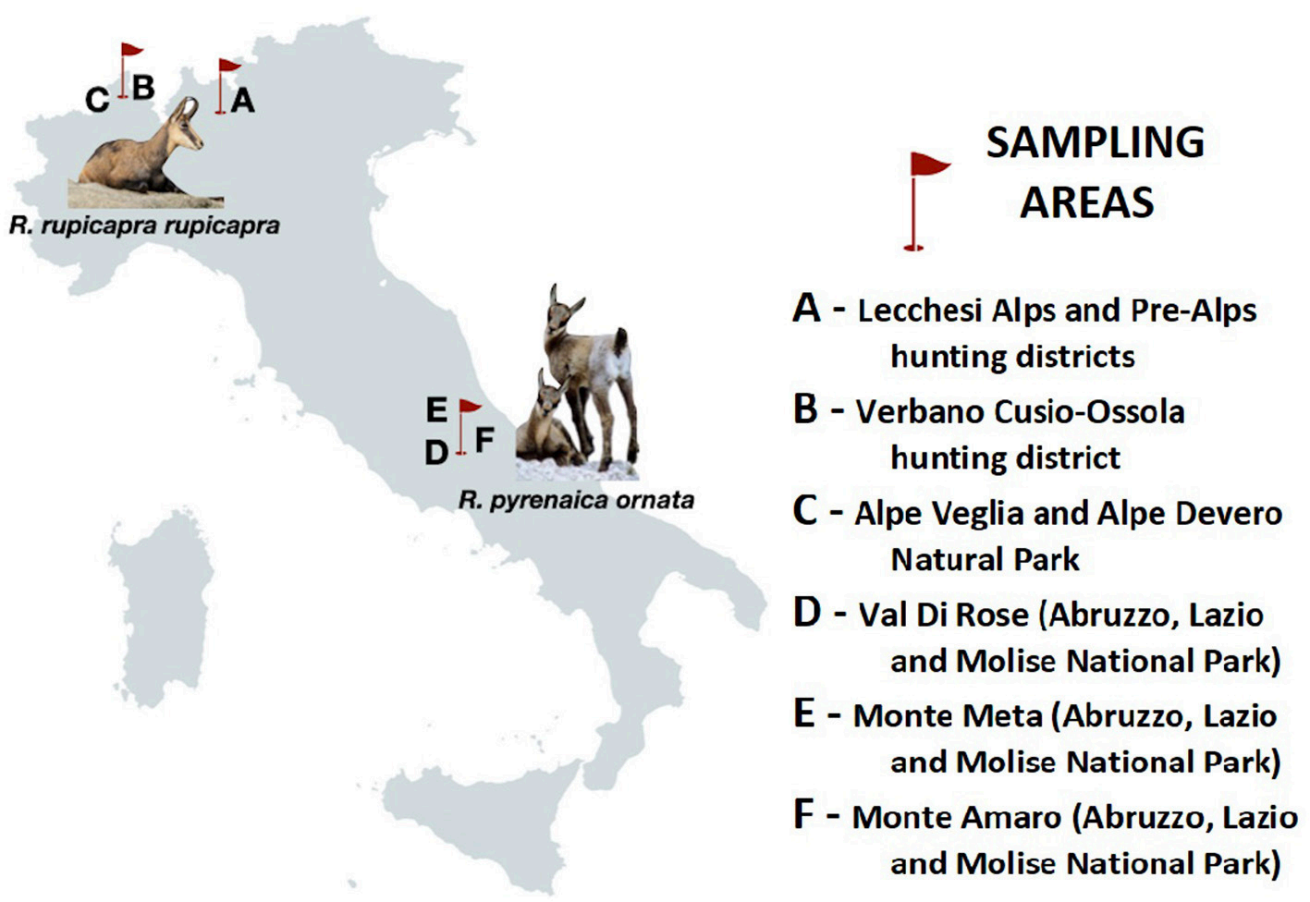
Schematic map of Italy showing the geographical location of sampling sites (A–F) and animal hosts analyzed in the present study.

From September 2013 to November 2015, fecal samples (*n* = 262) were collected from *R. r. rupicapra* in Italian Central Alps. The chamois originated from (i) a hunting territory in Lombardy region, with an area of 253 km^2^ (45°59'N, 9°32'E) (A); (ii) two contiguous areas in northern Piedmont (46°07'N, 8°17'E) with different population management: a hunting district (B), which extends over 727 km^2^ and a protected area (C) of 85, 39 km^2^ where hunting is banned (Figure 1), and fresh stool samples were collected from the ground soon after defecation. In the same period, fresh fecal samples of *R. p. ornata* (*n* = 90) were collected after observing defecation, from individuals grazing on upper grasslands, in three subareas of the Abruzzo, Lazio and Molise National Park (D to F, Figure 1). All samples were stored in 2.5% potassium dichromate in a 50-ml tight screw cap plastic tube under constant aeration for sporulation for a minimum of two weeks at room temperature until microscopical analysis.

*Eimeria* oocysts were recovered by flotation in saline solution (density 1,200), while quantitative analysis was performed using a McMaster technique, with a lower detection limit of 50 oocysts per gram of feces (o.p.g.) (15). Morphometrical and morphological features of oocysts and of sporocysts were used for species identification based on the description in Levine and Ivens (4). In particular, their shape, width and length, the oocyst color, rough or smooth wall, the presence/absence of the oocyst micropyle and/or cap, were carefully scrutinized and photographed using a Leica DMD108 microscope, equipped with an integrated camera and image analysis system, by 60 X objective lenses. All measurements are in micrometers (μm).

A statistical analysis with R software v 1.1.463 (FactoMine and factoextra packages) was performed to obtained data, using the Hierarchical Clustering on Principal Components (HCPC) approach on a dataset of 292 sporulated oocysts. The parameters used were length, width and the length/width ratio (Shape index, L × W) for both oocysts and sporocysts. We decided to employ the HCPC approach as it makes possible to combine the three standard methods used in multivariate data analyses (16): (i) principal component methods (PCA, CA, MCA, FAMD, MFA), (ii) hierarchical clustering, and (iii) partitioning clustering, particularly the k-means method. Furthermore, the HCPC analysis allows the characterization of clusters of specimens based on all characters and on subsets of characters, weighting all characters equally.

## Results

Overall, 85.3% (300/352) (95%, C.I. = 81.5–89.1) of samples were microscopically positive to *Eimeria* spp., with a mean intensity of up to 776 o.p.g. Prevalence in *R. r. rupicapra* was 81.2% (213/262) (95%, C.I. = 77.1–86.8), with a mean intensity of 380 o.p.g.; in *R. p. ornata* the prevalence was 94.4% (85/90) (95%, C.I. = 89.7–99.2), with a mean intensity of 1,093 o.p.g.

Based on morphological analysis of oocysts, three morphotypes attributable to *E. rupicaprae, E. riedmuelleri*, and *E. yakimoffmatschoulskyi* were detected in both chamois species. In addition, *E. suppereri* was recognized in *R. r. rupicapra* from Area B and, noteworthy, two small oocysts recovered from one chamois originated from Area A and consistent with the descriptions of *E. alpina* were identified. Due to the absence of sporulated forms, these two oocysts were excluded from the statistical analysis.

**Figure 2:**
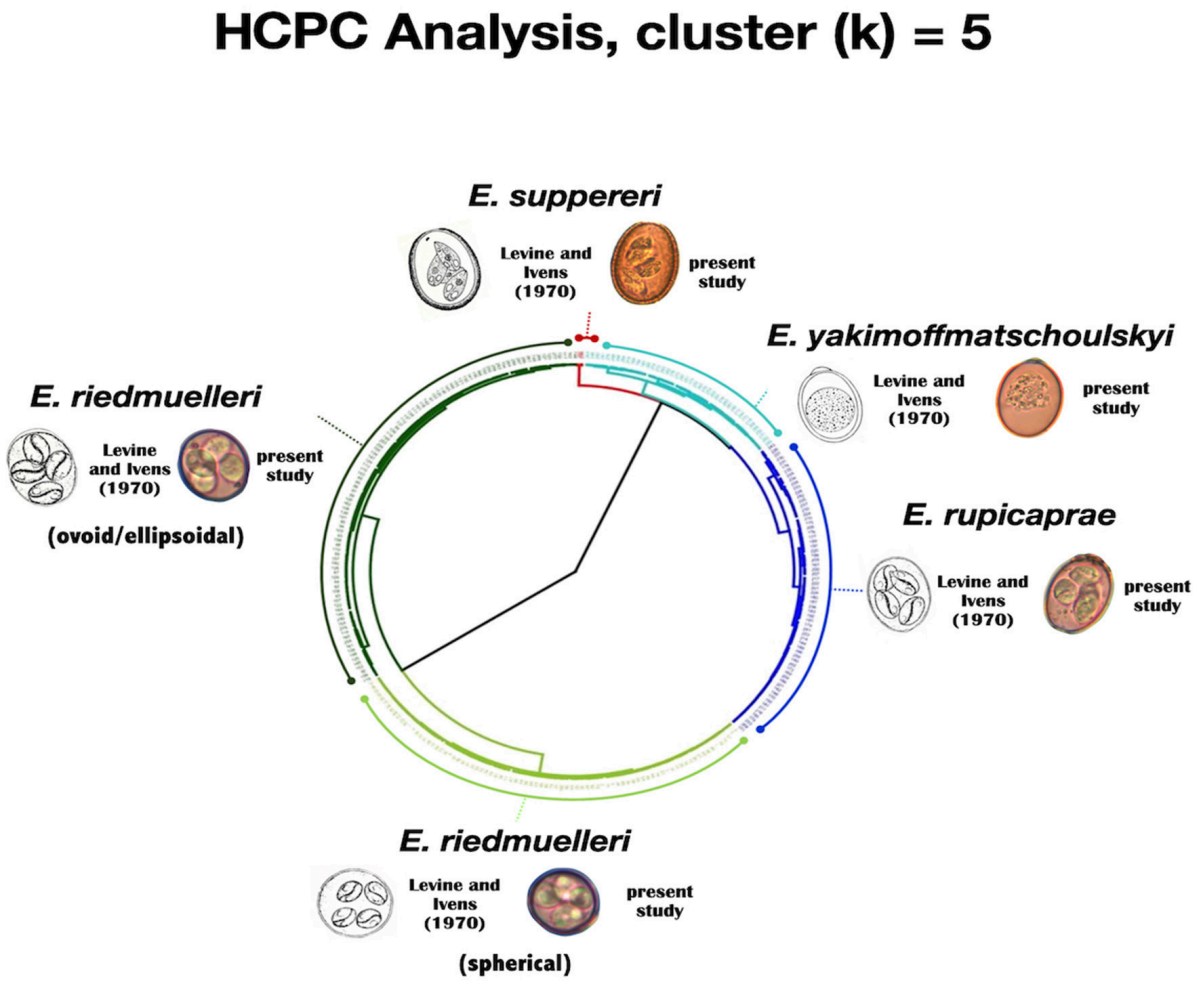
Circular dendrogram issued from the HCPC analysis (output of k = 5) based on length, width and shape index for both *Eimeria* spp. oocysts and sporocysts.

Through the HCPC analysis, five well-defined clusters (k = 5) grouping oocysts from both hosts were generated (see Table 1 and Figure 2). In summary, clustering was as follows:

**Table 1:** Morphological data of *Eimeria* oocysts and sporocysts isolated from Italian chamois (*Rupicapra* spp.).

**Species identification(Cluster)**	**Oocysts**			**Sporocysts**			**Host**
	**Length Mean Min-max**	**Width Mean Min-max**	**Shape index**	**Length Mean Min-max**	**Width Mean Min-max**	**Shape index**	
*E. suppereri* (Cluster 1)	47.54 46-49.08	35.38 34.5-36.26	1.34	19.48 17.5-21.47	10.88 10-11.76	1.79	*R. r. rupicapra*
*E. yakimoffmatschoulskyi* (Cluster 2)	29.71 25.25-35.97	21.3 18.14-25.64	1.39	13.74 8.9-17.01	6.92 5.2-8.66	1.98	*R. r. rupicapra R. p. ornata*
*E. rupicaprae* (Cluster 3)	27.02 22.24-32.64	22.33 18.5-27.50	1.21	11.75 7.01-17.4	7.71 5.9-11.13	1.5	*R. r. rupicapra R. p. ornata*
*E. riedmuelleri* Spherical form (Cluster 4)	18.28 14.63-22	16 14.25-20.78	1.13	7.57 5.93-9.66	5.99 4.67-7.18	1.26	*R. r. rupicapra R. p. ornata*
*E. riedmuelleri* Ovoid/ellipsoidal form (Cluster 5)	20.21 15.3-24.42	17.4 14.16-21.52	1.16	8.14 6.31-10.45	6.24 4.59-8.35	1.3	*R. r. rupicapra R. p. ornata*
*E. alpina* (-)	11.12 10.4-11.85	11 10.15-11.85	1	-	-	-	*R. r. rupicapra*

*Arithmetic mean of measurements of length, width and shape index, with minimum and maximum values are indicated for all Eimeria species analyzed in the present study. All measurements are in micrometers*.

Cluster 1 (red cluster) gathers the biggest oocysts (*n* = 4) of our dataset, measuring on average 47.54 by 35.38 μm; the mean measures of the sporocysts were 19.48 by 10.88 μm. These isolates were assigned to *E. suppereri*. Cluster 2 (light blue cluster) grouped 37 oocysts measuring on average 29.71 by 21.3 μm. The sporocysts measured 13.74 by 6.92 μm. We attributed these isolates to *E. yakimoffmatschoulskyi*. Cluster 3 (dark blu cluster) includes 75 oocysts measuring 27.02 by 22.33 μm. The sporocysts were 11.75 by 7.71 μm. We assigned these isolates to *E. rupicaprae*. Cluster 4 (light green cluster) consists of 82 spherical oocysts with a mean size of 18.28 by 16 μm. The sporocysts were 7.57 by 5.99 μm. We assigned these isolates to the spherical form of *E. riedmuelleri*. Finally, Cluster 5 (dark green cluster) pools 96 oocysts measuring on average 20.21 by 17.4 μm. The sporocysts were 8.14 by 6.24 μm. These isolates were assigned to the ovoid/ellipsoidal *E. riedmuelleri* form.

A detailed description of morphometrical data is given in Table 1.

## Discussion

Eimeriid protozoans are common parasites in ruminants worldwide, often associated with enteritis, weight loss and mortality in young animals (17). High prevalence and intensity of infection have been also documented in wild ungulates, where asymptomatic infection largely prevail (18). The high prevalence and intensity of oocysts emission recorded in this study shows that infection by eimeriid protozoa is also widespread amongst members of the *Rupicapra* genus in Italy, confirming previous findings (12, 19). The normal fecal consistency of analyzed samples suggests that infection by *Eimeria* spp. is substantially sub-clinical in both hosts.

To overcome the considerable amount of intraspecific and interspecific variation exhibited in the key morphological features of oocysts, and the drawbacks linked to the presence of multiple infections, as usually occurs in wildlife, in the present study a statistical method was performed for the identification of *Eimeria* spp. The hierarchical cluster analysis adopted here was able not only to verify the robustness of original taxonomic description of the *Eimeria* species known to parasitize the members of the genus *Rupicapra*, but, noteworthy, to provide a statistical significance to their morphological variability. Cluster analysis (see Figure 2) highlights that distinct forms can be separated based upon their morphology. As reported in Levine and Ivens (4), the measurements obtained from the oocysts grouped in Cluster 1 and 2 overlap unequivocally with the values describing *E. suppereri* and *E. yakimoffmatschoulskyi* according to Restani (11), respectively; Cluster 3, grouping oocysts of *E. rupicaprae* corresponds more strictly to Restani (11) measurements than to those by Galli-Valerio (20, 21) and Yakimoff and Matschoulsky (22). Surprisingly, within the species *E. riedmuelleri*, the splitting of the two well-defined Clusters 4 and 5 was in line with the two morphotypes (the spherical and the ovoid or ellipsoidal oocysts) described by Yakimoff and Matschoulsky (22) and Levine and Ivens (4). Hence, our results based on statistical method strongly support the existence of these distinct morphological groups, possibly referable to two different taxonomic entities infecting chamois, whose identity requires further in-depth investigations.

Remarkably, the Northern and Southern chamois shared most of the *Eimeria* species identified, suggesting that wild Caprines may be a suitable model to explore in depth the amplitude of the host specificity characterizing eimeriid protozoa (23, 24).

The absence of the large-sized, hence easy detectable, *E. suppereri* in the Southern chamois might reflect a possible effect of the life history of the Apennine subspecies of *R. pyrenaica ornata*, characterized by prolonged population bottlenecks (9, 25).

In conclusion, this study provides a deepening into the diversity of *Eimeria* species and highlights the not negligible prevalence of these coccidian protozoan infecting chamois in Italy. The presence of *E. rupicaprae, E. yakimoffmatschoulskyi, E. suppereri* and of *E. riedmuelleri* in *R. r. rupicapra* and of *E. riedmuelleri, E. rupicaprae* in *R. p. ornata* is confirmed. Moreover, *E. alpina* and *E. yakimoffmatschoulskyi* are additional species of the eimerian fauna of the Northern and Southern chamois. The combination of morphological data with a robust statistical method, as here proposed, represents a useful approach to infer the taxonomy and, consequently, to investigate the epidemiology of these protozoans with the due accuracy.

## Data Availability Statement

The raw data supporting the conclusions of this article will be made available by the authors, without undue reservation.

## Ethics Statement

Ethical review and approval was not required for the animal study because this research did not involve purposeful killing of animals. Fecal samples in hunting districts were gathered from chamois legally shot by hunters in accordance with the Italian Law (157 of 11/02/1992) which implies that hunters have to carry culled wild ungulates to the control centers where, for each subject, age, sex, the shooting area, and morpho-biometric measures are registered. Thus, no animals were killed specifically for this study.

## Author Contributions

FB, PL, LR, MMDF, SD'A, and AG participated in study activities and in drafting and revising the manuscript. CDL, IM, NF, TT, NF, and FF participated in the field and laboratory work. MMDF performed the statistical analysis. All Authors have participated in critically revising the manuscript.

## Conflict of Interest

The authors declare that the research was conducted in the absence of any commercial or financial relationships that could be construed as a potential conflict of interest. The handling Editor declared a past co-authorship with the authors SD'A.
